# The Structure of an RNAi Polymerase Links RNA Silencing and Transcription

**DOI:** 10.1371/journal.pbio.0040434

**Published:** 2006-12-05

**Authors:** Paula S Salgado, Minni R. L Koivunen, Eugene V Makeyev, Dennis H Bamford, David I Stuart, Jonathan M Grimes

**Affiliations:** 1 Division of Structural Biology, The Henry Wellcome Building for Genomic Medicine, Oxford University, Oxford, United Kingdom; 2 Institute of Biotechnology, Viikki Biocenter, University of Helsinki, Helsinki, Finland; 3 Department of Biological and Environmental Sciences, Viikki Biocenter, University of Helsinki, Helsinki, Finland; Brandeis University, United States of America

## Abstract

RNA silencing refers to a group of RNA-induced gene-silencing mechanisms that developed early in the eukaryotic lineage, probably for defence against pathogens and regulation of gene expression. In plants, protozoa, fungi, and nematodes, but apparently not insects and vertebrates, it involves a cell-encoded RNA-dependent RNA polymerase (cRdRP) that produces double-stranded RNA triggers from aberrant single-stranded RNA. We report the 2.3-Å resolution crystal structure of QDE-1, a cRdRP from *Neurospora crassa,* and find that it forms a relatively compact dimeric molecule, each subunit of which comprises several domains with, at its core, a catalytic apparatus and protein fold strikingly similar to the catalytic core of the DNA-dependent RNA polymerases responsible for transcription. This evolutionary link between the two enzyme types suggests that aspects of RNA silencing in some organisms may recapitulate transcription/replication pathways functioning in the ancient RNA-based world.

## Abstract 2

Insights into RNA-induced gene silencing are gained from the structure of an RNAi (RNA-dependent RNA) polymerase from*Neurospora crassa*. A striking resemblance to eukaryote and bacterial DNA-dependent RNA polymerases was observed.

## Introduction

RNA silencing, or RNA interference (RNAi), refers to a group of RNA-induced gene silencing mechanisms that developed early in the eukaryotic lineage and play essential roles in cellular immunity, modulation of chromatin structure, and development [1–3]. RNAi can induce transcriptional gene silencing (TGS) via chromatin repression or posttranscriptional gene silencing (PTGS) by degradation of target RNAs. RNA silencing pathways use double-stranded RNA (dsRNA) triggers, processed by Dicer, to form short interfering RNAs (siRNAs) of 21–25 nucleotides [4]. One siRNA strand is recruited by an effector complex containing the Argonaute protein and used as a guide for sequence-specific degradation of target mRNAs (in PTGS) or directed silencing of cognate chromatin domains (in TGS) [5,6]. A cell-encoded RNA-dependent RNA polymerase (cRdRP) is also involved, in plants, fungi, protozoa, and certain animals, but apparently not insects and vertebrates, producing dsRNA triggers and hence amplifying the PTGS response [7–9]. Furthermore, cRdRPs may also interact with the cellular transcription apparatus and effect chromatin silencing [10].

Transgene silencing in Neurospora crassa (quelling) is one of the best studied models for RNAi. Three genes products were identified by screening for quelling defective phenotype, QDE-1 (cRdRP), QDE-2 (Argonaute), and QDE-3 (RecQ-like helicase) [11]. Subsequent mechanism studies demonstrated the additional involvement of two Dicer-like proteins: DCL-1 and DCL-2 [12]. We have previously isolated recombinant QDE-1 and an enzymatically active C-terminal portion of the molecule (QDE-1 ΔN, residues 376–1,402), and have shown that QDE-1 efficiently produces full-length copies or short 9–21-nt copies scattered throughout the input ssRNA templates, as well as extending, rather inefficiently, complementary primers [9].

In order to understand the functionality of the QDE-1 in more detail, we have determined the high-resolution crystal structure. This reveals the molecule to be dimeric and containing at its core two subdomains responsible for catalysis. These subdomains each have the topology of double-psi β-barrels (DPBBs), and are similar to (and disposed in a similar fashion to) two separate subunits in the DNA-dependent RNA polymerases (DdRPs) that perform transcription across the domains of life. The structure not only suggests how the molecule might efficiently produce dsRNA triggers, but may also add a piece to the jigsaw that relates the RNA world to the DNA world, and provide a model for all cellular RdRPs.

## Results

### QDE-1 ΔN Is a Dimer with an Active Site Formed from DPBBs

**Figure 1:**
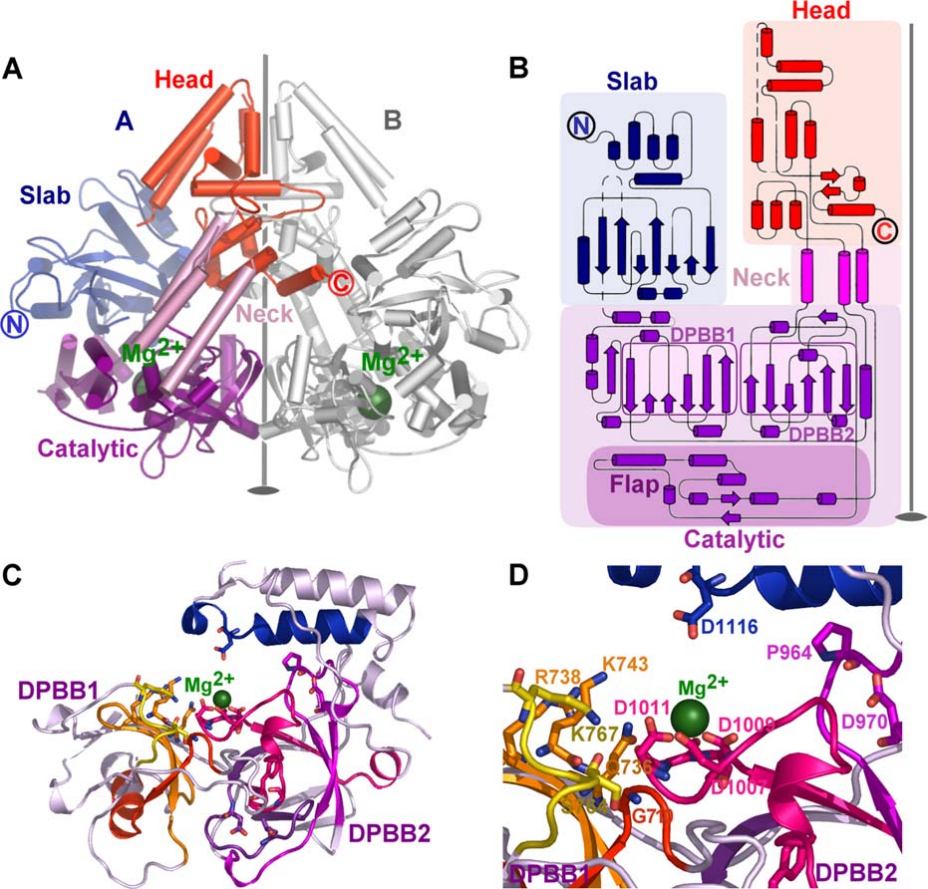
The Structure of QDE-1 ΔN (A) Cartoon representation of the QDE-1 ΔN *P2_1_* dimer–subunit A coloured according to domains: slab, blue; catalytic, purple; neck, pink; and head, orange; subunit B coloured grey. The approximate non-crystallographic 2-fold is represented as a grey line. Green spheres mark Mg^2+^ ions. Disordered regions correspond to ten residues at the N-terminus, 30 residues at the C-terminal, 45 residues in monomer A, and 48 in monomer B belonging to four loops (A: residues 590–603, 628–640, 1,241–1,251, and 1,271–1,281; B: 591–606, 627–640, 1,241–1,251, and 1,271–1,281). (B) Topology of QDE-1 ΔN subunit A, coloured as in (A). The catalytic subdomains DPBB1, DPBB2, and flap are denoted by boxes. The non-crystallographic 2-fold is represented as in (A). Disordered loops are represented by dashed lines. (C) View of presumed active site. The two DPBBs that form the active cleft, DPBB1 (residues 680–782) and DPBB2 (residues 916–1,018), are labelled. Sequence motifs conserved across other cRdRPs are highlighted: motif 1, red; 2, orange; 3, dark yellow, 4, purple; 5, dark pink; 6, bright pink; and 7, blue (see Figure S1). Invariant residues in cRdRPs are shown in ball-and-stick representation (O, red; and N, blue); Mg^2+^ ion shown as green sphere. (D) Zoom of active site region, with conserved residues labelled. Representation is as for (C).

The structure of QDE-1 ΔN was initially solved by multiwavelength anomalous diffraction (MAD) analysis of a crystal of selenomethionated protein, expressed in yeast, to a resolution of 3.2 Å [13]. The structure was then refined against a higher resolution dataset extending to 2.3 Å. These crystals belong to space group *P2_1_,* and the crystallographic asymmetric unit contains two subunits, which together form a compact pyramidal object with dimensions of 90 × 70 × 127 Å^3^. The two constituent subunits are related by an approximate 2-fold axis and are in such intimate contact (over 2,000 Å^2^ of contact area per subunit) that we would expect this to represent a functional dimer [14]. This was confirmed by gel filtration and sedimentation assays, with a molecular weight (MW) approximately equal to 230 kDa (compared to a predicted ∼120 kDa/monomer). QDE-1 ΔN has 1,026 residues, and the final model contains 933 residues in subunit A and 930 residues in subunit B (residues are missing from the N- and C-termini and from some flexible loops, see Figure 1). The refined structure at 2.3 Å has an R_work_ of 21.7% (R_free_ = 26.4%), and the stereochemistry is good (root mean square deviation [rmsd] bond = 0.013 Å, rmsd angle = 1.9°, 2.3% of residues are in disallowed regions of the Ramachandran plot; Table 1). In addition, a lower resolution structure (3.5-Å resolution) was determined in space group *C2* (see Table 1). In space group *C2,* the dimer is formed by two subunits related by exact crystallographic 2-fold symmetry. Both crystal forms use the same residues to stabilize the dimer, and these residues are primarily contributed by the head domains (head domain contacts account for 1,720/1,710 Å^2^ of the contact area of 2,215/2,100 Å^2^ per subunit in the *P2_1_*/*C2* space groups, respectively [15]).

**Table 1:**
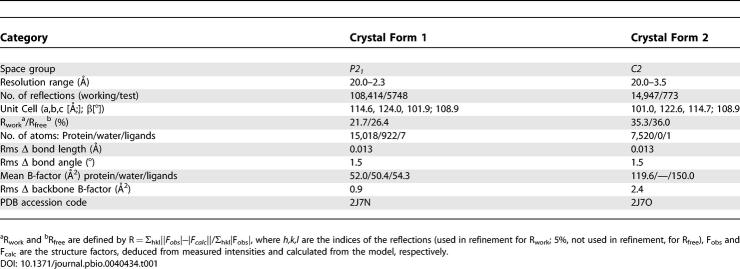
Refinement Statistics

Each QDE-1 subunit contains 41 α-helices and 25 β-strands, creating a four domain fold previously undescribed for RdRPs (Figure 1A and 1B). Distal to the molecular twofold axis is a mixed α/β “slab” domain composed of the approximately 250 N-terminal residues (390–646). The polypeptide chain then leads into a “catalytic” domain (residues 647–807; 914–1,161) that houses the three proposed catalytic aspartic acid residues [9] (D1007, D1009, and D1011; Figure 1C and 1D) within an active site cleft formed between two DPBB [16] subdomains (DPBB1, residues 690–792, and DPBB2, residues 916–1,018). The catalytic domain also contains a separate, mainly α–helical, “flap” subdomain (residues 1,025–1,161), peripheral to the active site cleft. The “neck” domain comprises three long α-helices (residues 808–836, 817–913, and 1,162–1,195), which lie close to the molecular twofold axis, connecting the catalytic domain to the mainly α-helical “head” domain (residues 837–888 and 1,196-1372; Figure 1A and 1B).

Multiple alignment of cRdRP amino acid sequences reveals the presence of seven motifs containing invariant residues: motifs 1–3 map to DPBB1, 4–6 to DPBB2, and 7 corresponds to α-helices 29 and 30 at the inner face of DPBB2 (Figures 1B, 1D, and S1). The conserved motifs therefore cohere in three dimensions, with at their heart the proposed catalytic aspartates that reside in a loop (residues 1,007–1,011, motif 6) in DPBB2 at the interface of the two β-barrels. Analysis of electron density maps showed a Mg^2+^ ion coordinated by these aspartic acid side chains (the functional significance of this is underscored by the observation that the QDE-1 polymerase activity is dependent on divalent cations [9]). DPBB1 contributes several positively charged residues to the active cleft, which include three invariant residues: Q736, K743 (motif 2), and K767 (motif 3). Together these establish a network of hydrogen bonds with water molecules, linking the two DPBB subdomains (Figure 1D). Although we have been unable to determine structures for complexes of QDE-1 with RNA and/or nucleotides (NTPs), we are able to infer functional aspects from the architecture of the uncomplexed molecule and its unexpected similarity to other polymerases, as discussed below.

**Figure 2:**
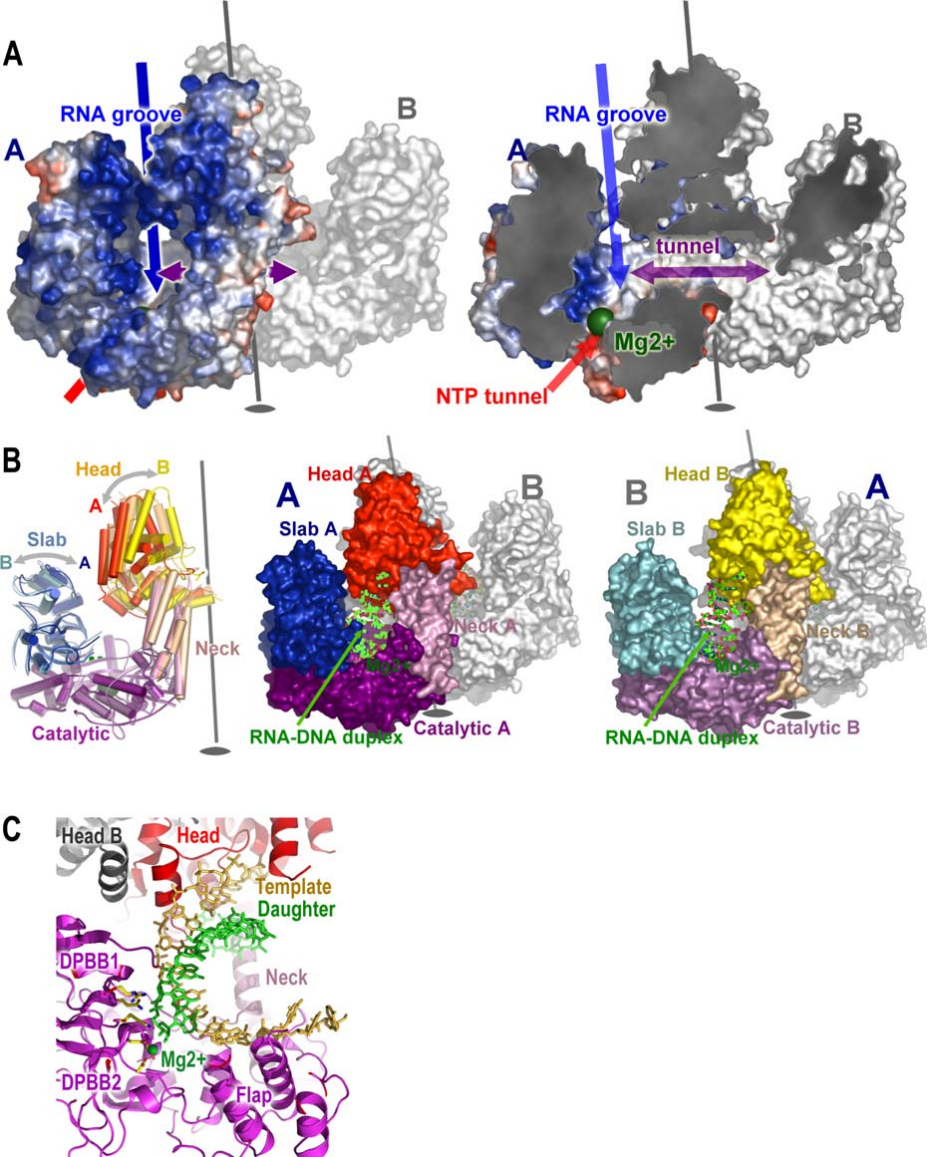
QDE-1 ΔN Surface Architecture (A) Surface charge representation of subunit A of QDE-1 ΔN (subunit B coloured grey). Blue arrow indicates proposed RNA product groove; red arrow indicates proposed NTP tunnel; and purple arrow indicates tunnel linking active sites in the dimer. The Mg^2+^ ions are shown as green spheres. Left panel: dimer (view similar to Figure 1A). Right panel: sliced view of left panel to reveal the tunnels. (B) Different conformations of QDE-1 ΔN subunits modify polymerase active site accessibility. Left panel: cartoon representation of superposition of observed subunit conformations, coloured according to domain definition: subunit A as in Figure 1A; subunit B: slab, cyan; catalytic, magenta; neck, wheat; and head, yellow; *C2* crystal form subunit (semi-transparent): slab, marine; catalytic, violet; neck, light salmon; and head, orange. In all panels, Mg^2+^ ions are represented as green spheres. The direction of the movement of the slab and head domains is indicated by grey arrows, and the non-crystallographic 2-fold is shown. Central and right panels: RNA-DNA duplex (from an elongation complex of yeast RNApolII) fitted (using the operators for superposition of yeast RNApolII onto QDE-1) into QDE-1 ΔN closed and open subunits (represented as molecular surfaces). Centre panel colours subunit A (closed conformation). Right panel shows a view rotated by 180° with subunit B (open conformation) coloured. RNA-DNA duplex model is show in ball-and-stick representation. (C) Close-up of the duplex model from (B). Domains are coloured as for the central panel of (B). The slab domain of molecule A would lie across the front of the figure and has therefore been removed for clarity.

The QDE-1 ΔN molecule has several distinct channels and cavities: first, there is a channel formed between the slab and head of each subunit, which is highly positively charged and leads to the active site (Figure 2A); we propose that these channels accommodate dsRNA product. Second, there is a small, negatively charged tunnel at the bottom of each subunit (formed between the flap and DPBB subdomains), which communicates with the active site and may be a route of entry for NTPs (Figure 2A). Third, there is a single tunnel, for which we cannot propose a function, formed between the neck domains and the catalytic domains, bridging the two active sites. The proposed dsRNA product binding channels are not identical in the two subunits, because the disposition of the domains is different: subunit A has a “closed” conformation, with the head and slab clamped down on the active site cleft, whereas an 11° rotation of the head and 2° rotation of the slab render the B subunit more open and provide space for an RNA duplex (Figure 2B and 2C). In the lower resolution *C2* crystal form, the molecule forms a symmetric dimer. Here, the subunits both assume a partially closed conformation (the head is rotated by 4° relative to A and 8° to B, and the slab is displaced upwards and outwards by 4° relative to A and 2° to B). Overall, QDE-1 exists as a compact, but flexible, dimeric enzyme with metal binding sites confirming positions of the catalytic sites but with extensive additional structure whose biological functions are less immediately clear.

### QDE-1 Active Site Is Closely Similar to Those of DdRPs

**Figure 3:**
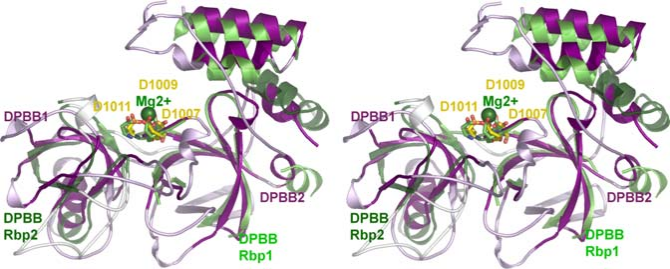
Comparison with DdRPs Stereo representation of the superposition of QDE-1 ΔN and yeast DdRP DPBBs (view as in Figure 1C). Structurally equivalent residues in QDE-1 ΔN are coloured dark purple (non-equivalent residues in light purple). Equivalent residues in yeast DPBBs are coloured green (non-equivalent residues in semi-transparent grey). QDE-1 ΔN and yeast (D481, D483, and D485) active site aspartates are coloured yellow and green respectively.

**Figure 4:**
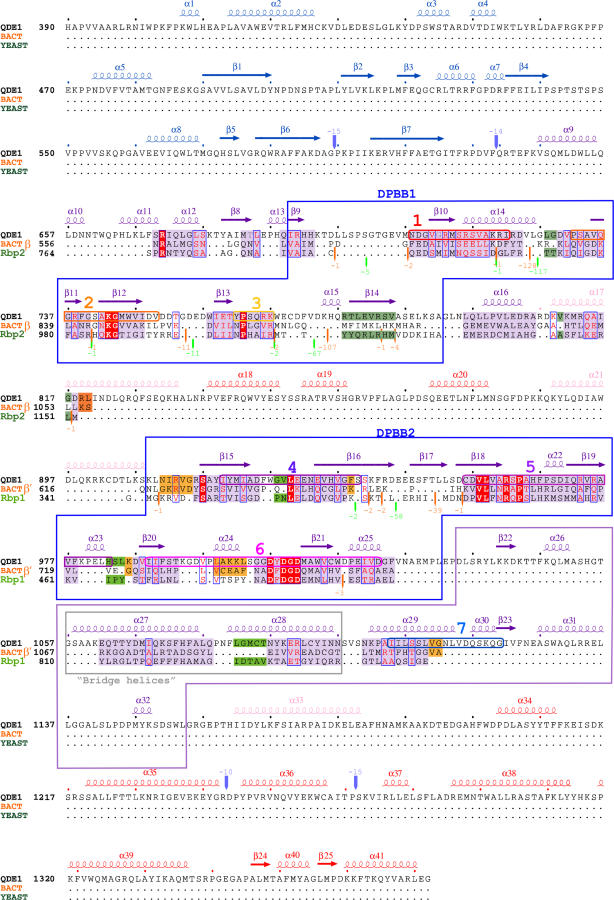
Structure-Based Sequence Alignment of QDE-1 ΔN and DdRPs Alignment of QDE1 ΔN (top sequence), bacterial (middle sequence, orange), and yeast (bottom sequence, green) polymerases is based on structurally equivalent residues, as determined by SHP [41]. Residues structurally equivalent in all polymerases are shaded purple, green (light for Rbp1, dark for Rp2) if equivalent in yeast DdRP and QDE1 ΔN, and orange if only equivalent in QDE1 ΔN and bacterial DdRP. Invariant residues are shaded in red. Conserved sequence motifs identified in cRdRPs are represented as in Figure S1, marked on QDE1 sequence. QDE1 secondary structure elements are shown on top, coloured according to domain definition (slab, blue; catalytic, deep purple; neck, pink; and head, red). DPBB1 and DPBB2 are outlined by deep purple boxes. The flap sub-domain and the potential “bridge helix” are also represented by boxes, coloured light purple and grey, respectively.

A search using the QDE-1 ΔN active site residues (ASSAM [17]) identified similarities with the yeast [18] and bacterial [19] DdRPs. Superposition revealed that the DPBB subdomains in QDE-1 and the DdRPs are structurally very similar and almost identically disposed (∼10° change in the relative positions of the subdomains; Figure 3). The DdRPs have a DPBB in each of two largest subunits (β′ and β subunits in the bacterial enzyme, and Rbp1 and Rbp2 subunits in yeast RNA pol II), with the first contributing the catalytic aspartates to the active site and the other a set of positively charged residues [18]. In QDE-1 ΔN, the two subdomains have a similar segregation of chemical roles, but they are arranged sequentially on a single polypeptide chain. The similarity of the DPBB bearing the catalytic aspartates between the cRdRPs and DdRPs had been predicted from sequence analysis [20]; however, there is no sequence homology detectable in the second DPBB or elsewhere in the molecule (see Figure 4). Superposition matches 81 residues of DPBB2 in QDE-1 ΔN with the bacterial β′ DPBB and 85 residues with the yeast Rbp1 DPBB (2.2 and 2.1 Å rmsd in Cαs, respectively). DPBB1 is somewhat less similar to the homologous domain in the DdRPs (74 and 67 residues matched with rmsd of 3.0 and 3.1 Å for bacterial and yeast, respectively; Figure 3). The QDE-1 ΔN catalytic aspartates lie within 1.4 Å of the bacterial and yeast catalytic residues, with equivalent Mg^2+^ coordination to metal A in the yeast enzyme [18]. Moreover, the bridge helices in DdRPs, proposed to be important for nucleic acid–protein interactions during translocation of the duplex [18,21], are structurally equivalent to helices 27 and 28 in QDE-1 ΔN, suggesting a similar role. Superposition onto QDE-1 of the structure of yeast DdRP complexed with an RNA–DNA duplex [21] maps the duplex into the putative RNA product groove in QDE-1 (Figure 2B) and the proposed NTP tunnel matches well with that proposed in yeast RNA pol II [22]. Intriguingly, only about ten base pairs of duplex RNA can be modelled into this groove without severe steric clashes with the head domain, suggesting a steric basis for modulating the length of RNA synthesised (Figure 2C).

Overall, the DdRP structures are much more elaborate than QDE-1 ΔN, and we can detect no significant structural similarity beyond the vicinity of the active site. Nevertheless, there may be some functional relationship at the level of protein domains. The flexible head that forms part of the proposed QDE-1 product groove might be equivalent to the clamp in yeast Rbp1 [18]: the head domain closing down on the slab to stabilize the RNA product during polymerisation (Figure 2B). The yeast Rbp2 protrusion-lobe is also displaced during transcription [18] to accommodate and stabilize the RNA–DNA product, and the somewhat flexible QDE-1 slab may play a similar role. These observations suggest a similar mode of action for the DdRP and cRdRPs, a view reinforced by the proposal that a human DdRP, Pol II, is involved in replication of hepatitis Δ RNA [23].

## Discussion

### QDE-1 May Act as a Two-Stroke Motor for the Production of dsRNA Triggers

QDE-1 ΔN is active in solution [9], and we have shown here that this form is dimeric. What, if anything, might the functional significance of the dimer be? Three roles for cRdRPs have been discussed: (1) conversion of aberrant RNAs into dsRNA triggers for PTGS, (2) primer-dependent amplification of RNAi triggers, and (3) chromatin silencing [2,8–10]. Reaction (1) presumably involves initiation at the 3′ end of aberrant mRNA and processive RNA synthesis, whereas reactions (2) and (3) would require internal recruitment of the polymerase to its RNA targets. Both initiation modes function in vitro [9,24]. The more open conformation of subunit B would allow internal initiation, but conversely, the partial closure of the RNA product groove in the dimer might favour initiation at the 3′ end of an RNA template. It seems likely that, in the dimer, only one catalytic site is active at any given time, represented by the closed conformation of subunit A, with the inactive subunit being held open. This suggests a mechanism whereby binding to one active site primes the other. Thus, the molecular architecture might favour the molecule working as a “two-stroke motor,” with facilitated active site switching as the subunits cycle back and forth between conformations in response to RNA binding. This model has two attractive features: first, by tethering the RNA template to the dimer, re-initiation will be efficient, and second, initiation at active site B can be coupled to the activity at active site A, by molecular switching, driven by the steric clashes with the rather stiff dsRNA product suggested by our modelling studies. Overall, this mechanism might lead to the effective production of appropriate-length dsRNA triggers.

### A Polymerase for the RNA World

**Figure 5:**
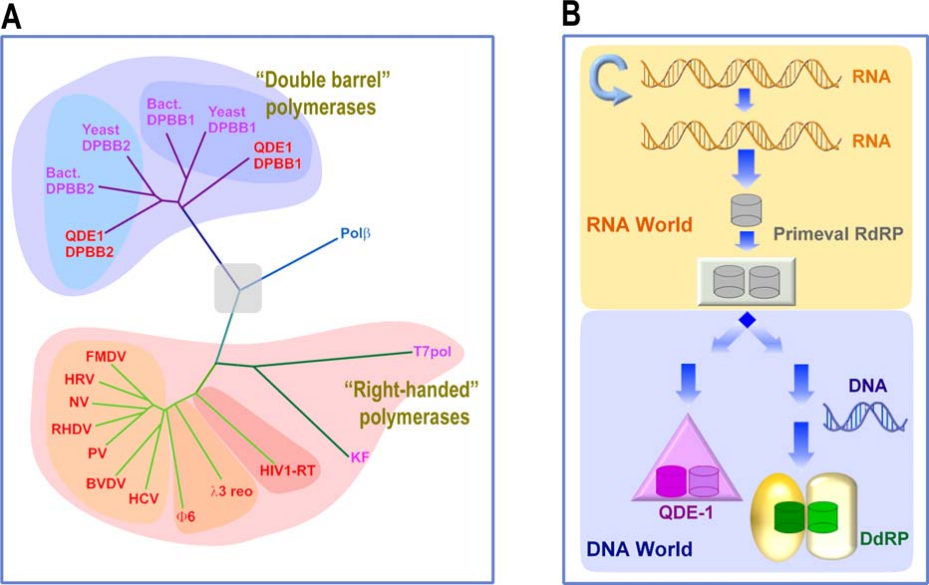
Evolutionary Relationships (A) Evolutionary phylogenetic tree for known polymerases based on structural similarity (for description of the method see [43]). The “right-handed” polymerases, “double-barrel” polymerases, and polβ appear to form three separate, unrelated families. Within the “double-barrel” family, the DPBB2 domains containing the active site aspartate residues are more structurally conserved than DPBB1. Branches are coloured according to structural fold: green, right hand (dark, cellular; and light, viral), Polβ, blue; and DPBB-containing fold, dark magenta. Since we do not believe that all polymerases originate from a common ancestor, the central node of the tree is shaded grey. The key to the additional structures is: Yeast DPBB1, yeast RNApolII DPBB from Rbp2; Yeast DPBB2, yeast RNApolII DPBB from Rbp1; Bact. DPBB1, bacterial β subunit DPBB; Bact. DPBB2, bacterial β′ subunit DPBB; Polβ, rat DNA polymerase β; T7pol, bacteriophage T7 DdRP; KF, Klenow fragment of DNA polymerase I; HIV1-RT, human immunodeficiency virus type 1 (HIV-1) reverse transcriptase (RT); λ3 reo, reovirus RdRP; Φ6, Φ6 bacteriophage RdRP; HCV, hepatitis C virus RdRP; BVDV, bovine viral diarrhoea virus RdRP; PV, poliovirus RdRP; RHDV, rabbit hemorrhagic disease virus RdRP; NV, Norwalk virus RdRP; HRV, human rhinovirus RdRP; and FMDV, foot–and-mouth disease virus RdRP. (B) Originally, in an all RNA world, RNA self-replicates until the advent of a protein-based, primeval RNA-dependent RNA polymerase. Initially this possesses a single DPBB domain on a single polypeptide chain. Gene duplication leads to a polypeptide chain containing two copies of the DPBB domain. Differentiation of the two DPBB domains then results in QDE-1–like RdRPs. Emergence of DNA and associated increase of complexity lead to segregation of the DPBB into different polypeptidic chains, giving rise to the complex multi-subunit DdRP machinery observed today.

From the structure of QDE-1, it appears that all cellular RdRPs as well as DdRPs are related but structurally distinct from viral RdRPs, which have the “right-hand” architecture [25–30]. The similarity revealed here between the active sites of cRdRPs and the DdRPs indicates that they share a common ancestor. We refer to this family of enzymes as the “double-barrel” polymerases. The RNAi polymerases present the entire active site on a single polypeptide chain, suggesting that they are closer to the ancestral protein than the present day transcription polymerases, which are composed of up to 12 separate polypeptide chains, and in general bear the two active site β-barrels on separate chains. Interestingly the DdRP of Helicobacter pylori also has its active site on a single chain, due to the fusion of the *rpoB* and *rpoC* genes, coding for the β and β′ subunits [31]. Indeed, it has been shown in vitro that a fused Escherichia coli β-β′ protein can assemble into a functional polymerase, although there is presumably some advantage in vivo for separate β and β′ chains [32]. Classifying the evolutionary relatedness of polymerases on the basis of their structural similarity, reveals that the “right-handed” and “double-barrel” polymerases are tightly grouped and quite separate from each other and also from Polβ (Figure 5A). The implied path for the evolution of the double-barrelled RNA polymerases is shown in Figure 5B. The original enzyme presumably possessed a single DPBB, bearing the essential catalytic apparatus. This molecule may have taken over the role of RNA polymerisation, presumably from an RNA molecule, very early in evolution. Gene duplication led to a molecule with two DPBBs on a single polypeptide chain, which differentiated to produce a QDE-1–like molecule capable of efficient RNA polymerization, a key development in the elaboration of the RNA-based world. During this process, DPBB2, containing the active site aspartate residues, was conserved relatively strongly, whereas DPBB1, which acts as an accessory domain, essentially lost any detectable sequence conservation (Figure 4). The ability of such molecules to act on DNA could then underpin the switch to DNA as the repository of genomic information. Splitting of the DPBBs onto separate subunits would facilitate the radical evolution of this first DdRP into the complex, highly regulated, transcription machines with particular functions delegated to specialist subunits, which we observe today. In summary, it seems that this polymerase part of the RNA silencing machinery may give us a glimpse far back in time, providing insight into the evolution of a protein-based mechanism for the transmission of RNA genomic information in an RNA-based world.

## Materials and Methods

### Structure determination.

The protein expression in yeast, purification, crystallization, and data collection of a cryo-cooled selenomethionated QDE-1 ΔN crystal (space group *P2_1_*), and two native datasets (space group *P2_1_* and *C2*) have been described previously [13]. Briefly, a three-wavelength MAD experiment was performed using a cryo-cooled selenomethionated crystal of QDE-1 ΔN, to a resolution of 3.2 Å, on the Medical Research Council (MRC) MAD beamline, BM14, at the European Synchrotron Radiation Facility (ESRF) (Grenoble) using a MarCCD detector (Mar USA, Evanston, Illinois, United States). Subsequently, a native dataset to 2.3-Å resolution was collected on BM14 using a MarMosaic 225 CCD detector (Mar USA). The datasets from the three-wavelength MAD experiment were scaled and merged together and used in SnB [33] to solve the selenium substructure. The top 54 Se sites from SnB were refined using SOLVE [34] to obtain initial phases, and anomalous difference Fourier maps allowed the identification of a subset of 46 correct Se atoms that were refined using SHARP [35]. Phase improvement using density modification with RESOLVE [34] led to maps which, in combination with Se positions, revealed two articulated subunits, related by two slightly displaced non-crystallographic twofold axes, which precluded simple averaging. The initial RESOLVE model was completed using manual model building in O [36] and Coot [37], and automated model building and water placement with ARP/wARP [38]. The model was refined with REFMAC5 [39], using TLS refinement and imposing non-crystallographic restraints for the core regions of subunits, against the native *P2_1_* data to 2.3-Å resolution to yield the final model described in the main text and Table 1. The *C2* crystal form with one molecule in the asymmetric unit was solved by molecular replacement and refined, keeping the domains as rigid bodies (AMORE [40], Table 1).

### Structural alignments.

The program ASSAM [17] was used to find structural similarities with the proposed three catalytic aspartates and the coordinating magnesium ion. Structures of DNA-directed RNA polymerase II largest subunit were amongst the best matches. Superimposition operators for yeast and bacterial DdRP models onto to QDE-1 ΔN were optimized using SHP [41].

### Figures.

Figures were prepared using PyMOL (http://www.pymol.org) and ESPript [42].

### Oligomeric state of QDE-1 ΔN.

QDE-1 ΔN size was determined by (1) gel filtration (Superdex 200 16/60, equilibrated with 25 mM Tris-HCl [pH 8.0], 200 mM NaCl) with blue dextran (2,000 kDa), catalase (232 kDa), aldolase (158 kDa), bovine serum albumin (68 kDa), ovalbumin (43 kDa), chymotrypsin A (25 kDa), and RNase A (13.7 kDa) as molecular mass markers; and (2) by sedimentation in a linear 10% to 40% sucrose gradient in 50 mM Tris-HCl (pH 8.0), 350 mM NaCl (Sorvall TH641 rotor [Thermo Electron Corporation, Waltham, Massachusetts, United States], 35,000 rpm, 42 h, 15 °C), with catalase (232 kDa), bovine serum albumin (68 kDa), phage PM2 protein P2 (30.2 kDa), and lysozyme (14 kDa) as molecular mass markers.

## Supporting Information

Figure S1 Conserved Sequence Motifs in Cellular RdRPs Multiple sequence alignment of a representative subset of cRdRPs. Amino acid sequences of 30 cRdRPs from fungi from the groups of Ascomycota (*Schizosaccharomyces pombe,* Spo; *Neurospora crassa,* Ncr; and *Gibberella zeae,* Gze) and Basidiomycota (*Cryptococcus neoformans,* Cne), slime molds (*Dictyostelium discoideum,* Ddi), dicot plants (*Arabidopsis thaliana,* Ath; *Solanum tuberosum,* Stu; and *Nicotiana tabacum,* Ntu), monocot plants (*Oryza sativa,* Osa), protozoa (*Entamoeba histolytica,* Ehi), and nematodes (*Caenorhabditis elegans,* Cel) were aligned using standard settings of ClustalW algorithm. Local alignment was improved by manual editing. N. crassa QDE-1 protein sequence is shown on the top. N. crassa contains two additional non-allelic cRdRP genes—*SAD-1* (essential for meiotic silencing by unpaired DNA ) and *RdRP-3*—that likely function in distinct cellular pathways. Invariant residues are shaded in black; other residues with 80% or more conservation are shaded in grey. Conserved sequence motifs comprising invariant residues are outlined: motif 1, red; motif 2, orange; motif 3, dark yellow; motif 4, purple; motif 5, violet; motif 6, light pink; and motif 7, blue. QDE-1 secondary structure elements are shown on top, coloured according to domain definition (slab, blue; catalytic, deep purple; neck, pink; and head, red). The identified double-psi β-barrels DPBB1 and DPBB2 are outlined by deep purple boxes. The flap sub-domain and the potential “bridge helices” are also represented by boxes, coloured light purple and grey, respectively.
(914 KB DOC)

### Accession Numbers

Coordinates and structure factors have been deposited in the Protein Data Bank (PDB; http://www.rcsb.org/pdb) as accession numbers 2J7N and 2J7O. The GenPept accession numbers for the genes and gene products mentioned in this paper are N. crassa QDE-1 protein sequence (EAA29811); *dRP-3* (EAA34169); and *N. crassa SAD-1* (AAK31733).

## Abbreviations

cRdRP: cell-encoded RNA-dependent RNA polymerase
DdRP: DNA-dependent RNA polymerase
DPBB: double-psi β-barrel
dsRNA: double-stranded RNA
MAD: multiwavelength anomalous diffraction
NTP: nucleotide
PTGS: posttranscriptional gene silencing
rmsd: root mean square deviation
RNAi: RNA interference
TGS: transcriptional gene silencing
