# Supplementary material for: The Structure of an RNAi Polymerase Links RNA Silencing and Transcription
Source: PLoS Biol. 2006 Dec 5;4(12):e434. doi: 10.1371/journal.pbio.0040434 (PMC1750930; doi:10.1371/journal.pbio.0040434)
Supplement: Figure S1 — Multiple sequence alignment of a representative subset of cRdRPs. Amino acid sequences of 30 cRdRPs from fungi from the groups of Ascomycota (Schizosaccharomyces pombe, Spo; Neurospora crassa, Ncr; and Gibberella zeae, Gze) and Basidiomycota (Cryptococcus neoformans, Cne), slime molds (Dictyostelium discoideum, Ddi), dicot plants (Arabidopsis thaliana, Ath; Solanum tuberosum, Stu; and Nicotiana tabacum, Ntu), monocot plants (Oryza sativa, Osa), protozoa (Entamoeba histolytica, Ehi), and nematodes (Caenorhabditis elegans, Cel) were aligned using standard settings of ClustalW algorithm. Local alignment was improved by manual editing. N. crassa QDE-1 protein sequence is shown on the top. N. crassa contains two additional non-allelic cRdRP genes—SAD-1 (essential for meiotic silencing by unpaired DNA ) and RdRP-3—that likely function in distinct cellular pathways. Invariant residues are shaded in black; other residues with 80% or more conservation are shaded in grey. Conserved sequence motifs comprising invariant residues are outlined: motif 1, red; motif 2, orange; motif 3, dark yellow; motif 4, purple; motif 5, violet; motif 6, light pink; and motif 7, blue. QDE-1 secondary structure elements are shown on top, coloured according to domain definition (slab, blue; catalytic, deep purple; neck, pink; and head, red). The identified double-psi β-barrels DPBB1 and DPBB2 are outlined by deep purple boxes. The flap sub-domain and the potential “bridge helices” are also represented by boxes, coloured light purple and grey, respectively. (914 KB DOC) [file pbio.0040434.sg001.doc]

**Supporting Information**

The figure shows a multiple sequence alignment of a representative subset of cell-encoded RdRPs, with *N. crassa* QDE-1 protein sequence highlighted. Conserved sequence motifs comprising invariant residues, secondary structure for QDE-1 and structurally relevant sub-domains are outlined.


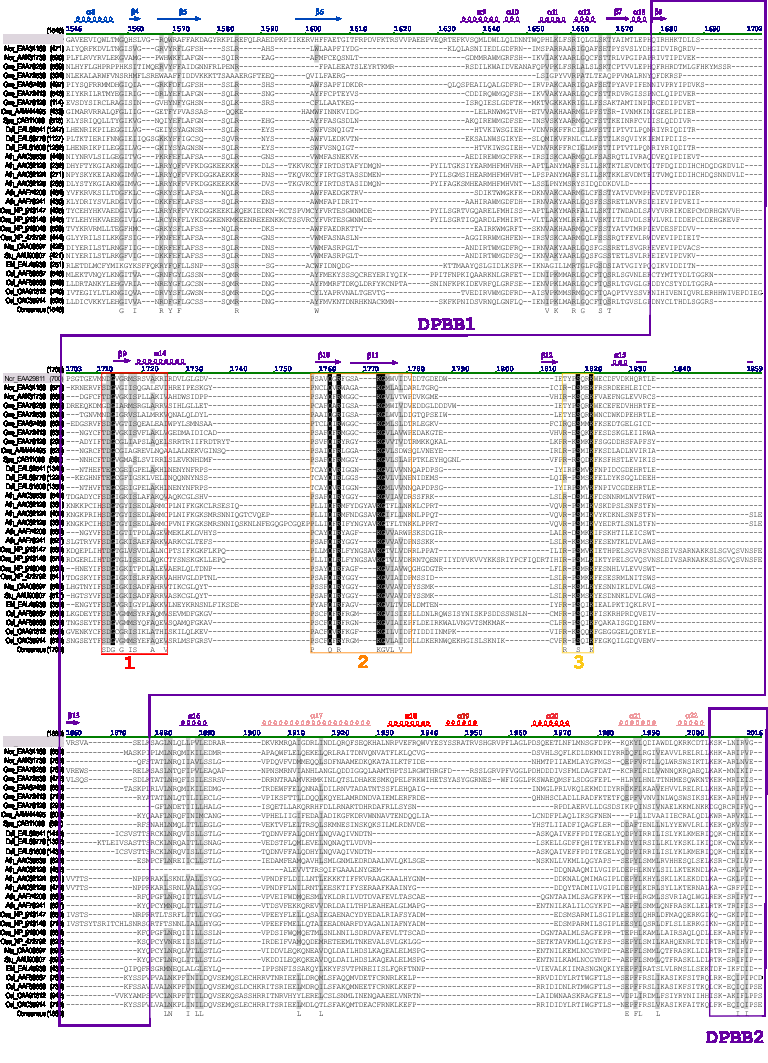
**Figure S1**

**
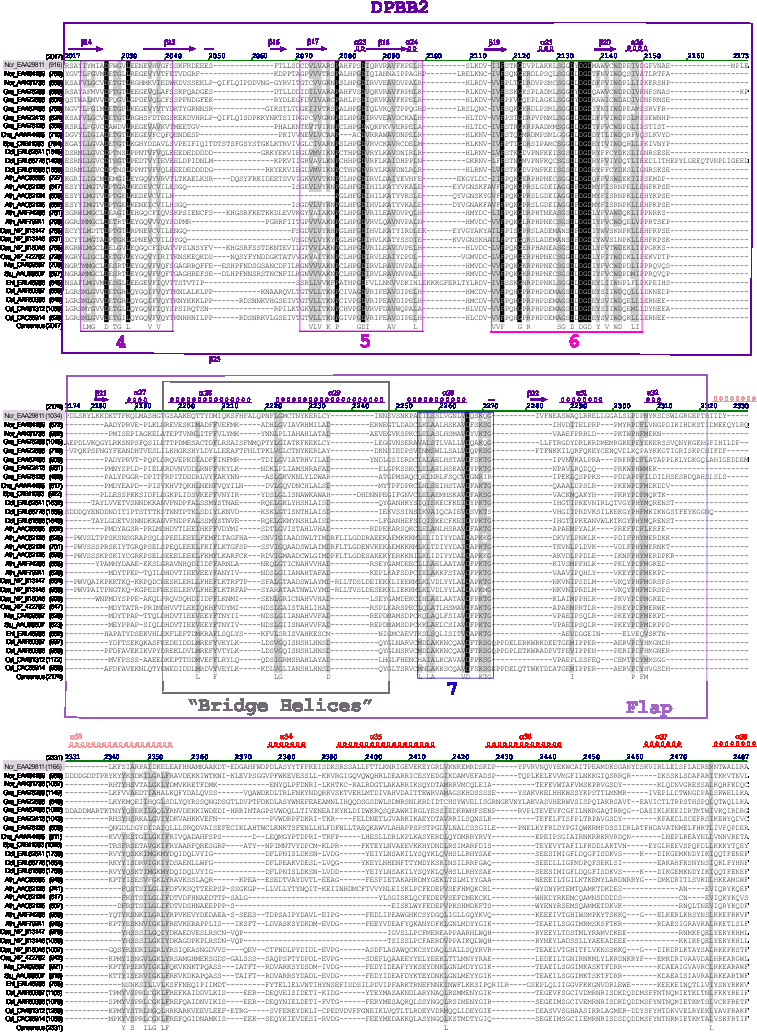
**

**Figure S1. Conserved sequence motifs in cellular RdRPs.** Multiple sequence alignment of a representative subset of cell-encoded RdRPs. Amino acid sequences of 30 cRdRPs from fungi from the groups of Ascomycota (*Schizosaccharomyces pombe*, Spo; *Neurospora crassa*, Ncr; *Gibberella zeae*, Gze) and Basidiomycota (*Cryptococcus neoformans*, Cne), slime molds (*Dictyostelium discoideum*, Ddi), dicot plants (*Arabidopsis thaliana*, Ath; *Solanum tuberosum*, Stu; *Nicotiana tabacum*, Ntu), monocot plants (*Oryza sativa*, Osa), protozoa (*Entamoeba histolytica*, Ehi), and nematodes (*Caenorhabditis elegans*, Cel) were aligned using standard settings of ClustalW algorithm. Local alignment was improved by manual editing. *N. crassa* QDE-1 protein sequence (accession number EAA29811) is shown on the top. *N. crassa* contains two additional non-allelic cRdRP genes - SAD-1 (accession number AAK31733) (essential for meiotic silencing by unpaired DNA ) and RdRP-3 (accession number EAA34169) - that likely function in distinct cellular pathway. Invariant residues are shaded in black; other residues with ≥80% conservation are shaded in grey. Conserved sequence motifs comprising invariant residues are outlined: motif 1 – red; motif 2 – orange; motif 3 – dark yellow, motif 4 – purple; motif 5 – violet; motif 6 – light pink; motif 7 – blue. QDE-1 secondary structure elements are shown on top, coloured according to domain definition (slab – blue; catalytic – deep purple; neck – pink; head – red). The identified double psi-β barrels DPBB1 and DPBB2 are outlined by deep purple boxes. The flap sub-domain and the potential “bridge helices” are also represented by boxes, coloured light purple and grey, respectively.
